# Bringing the state into the clinic? Incorporating the rapid diagnostic test for malaria into routine practice in Tanzanian primary healthcare facilities

**DOI:** 10.1080/17441692.2015.1091025

**Published:** 2015-10-12

**Authors:** Eleanor Hutchinson, Hugh Reyburn, Eleanor Hamlyn, Katie Long, Judith Meta, Hilda Mbakilwa, Clare Chandler

**Affiliations:** ^a^ Department of Global Health and Development, London School of Hygiene and Tropical Medicine, London, UK; ^b^ Department of Disease Control and Vector Biology, London School of Hygiene and Tropical Medicine, London, UK; ^c^ Joint Malaria Programme, Kilimanjaro Christian Medical Centre, Moshi, Tanzania

**Keywords:** Rapid diagnostic tests, malaria, complex intervention trials, Tanzania, targeted treatment

## Abstract

The roles that rapid, point-of-care tests will play in healthcare in low-income settings are likely to expand over the coming years. Yet, very little is known about how they are incorporated into practice, and what it means to use and rely upon them. This paper focuses on the rapid diagnostic test for malaria (mRDT), examining its introduction into low-level public health facilities in Tanzania within an intervention to improve the targeting of costly malaria medication. We interviewed 26 health workers to explore how a participatory training programme, mobile phone messages, posters and leaflets shaped the use and interpretation of the test. Drawing on notions of biopolitics, this paper examines how technologies of the self and mechanisms of surveillance bolstered the role mRDT in clinical decision-making. It shows how the significance of the test interacted with local knowledge, the availability of other medication, and local understandings of good clinical practice. Our findings suggest that in a context in which care is reduced to the provision of medicines, strict adherence to mRDT results may be underpinned by increasing the use of other pharmaceuticals or may leave health workers with patients for whom they are unable to provide care.

## Introduction

The lack of diagnostic tools in Africa has been identified as a central deficiency in the provision of healthcare on the continent (Okeke, 2011). Investment in developing new diagnostic tools is seeking to shift this trend and rapid point-of-care (POC) tests provide particular appeal. Easy to use, they are expected to enable swift diagnosis, without the necessity of significant investment in training, equipment or infrastructure associated with the development of laboratories (English et al., 2014; Jani & Peter, 2013; Mabey et al., 2012; Peeling & Mabey, 2010). POC tests are currently available for a number of infections (HIV, malaria and chlamydia, syphilis, typhoid) and others are expected to come into use over the next decade (Jani & Peter, 2013).

This paper is concerned with the introduction of the rapid diagnostic test for malaria (mRDT). Historically, malaria was treated presumptively but research published in 2004 raised concerns that this was leading to its over-diagnosis, the over-prescription of malaria medication, and the under-diagnosis of other infections (Reyburn et al., 2004). The introduction of a new, first-line therapy, artemisinin combination therapy (ACT), made the introduction of targeted treatment appear more urgent. ACTs are costly drugs but also in need of protection from emerging drug resistance, considered by some to be promoted by the over-prescription of medicine (Aung et al., 2015). The introduction of a highly accurate, easy-to-use rapid diagnostic test appeared as a practical solution to urgent problems of poor case management, increasing costs of medication, long-term underinvestment in laboratories and anti-microbial resistance.

Despite their considerable promise in global policy discourse, the results of the initial studies of the introduction of mRDTs in clinical settings in Africa were marked by disappointment. Tests were often left unused and on the occasions when they were carried out, patients with a negative test result were often treated for malaria (Bisoffi, Gobbi, Angheben, & Van den Ende, 2009; Reyburn et al., 2007). With much resting on the promise of the test to reduce the use the ACTs, a number of different training strategies were implemented and evaluated under trial conditions to try to increase the uptake of the mRDT and promote its use in providing a definitive diagnosis of malaria (Odaga et al., 2014). Several studies on their introduction have focused on the extent to which health workers will rely on mRDTs to guide decision-making through elicitation of an ‘adherence’ to guidelines outcome (Ansah et al., 2010; Mbacham et al., 2014). Little is known, however, about the significance that these tests hold for daily practices within clinics beyond their ability to direct (or not) the use of medication.

This paper provides an account of the effects of the tests on everyday practices in primary care settings in Tanzania as they were implemented during the Targeting Artemisinin Combination Therapy (TACT) intervention trial. Through a bio-political lens, the paper explores the work entailed in adhering to test results and the new regimes of treatment that they imply. It analyses the ways in which changes in prescribing practices were managed in the context of pre-existing socio-material relationships and moral frameworks in the clinic; and how powerful dilemmas could emerge when new policy went against understandings of good care already in place.

## Conceptualising mRDTs

In much of the literature on mRDTs, the test is conceptualised as an ‘autonomous determining force’ (Schubert, 2011) with a single role to play in the clinical encounter and with the impact of its introduction distinguishable by its intended and unintended consequences. In this paper, we shift the focus to incorporate the broad and sometimes contradictory effects of the test as part of its emergent, relational nature as a (health) tool, the significance of which is ultimately contingent upon the objects, people and social relations within which it is embedded (Rock, Degeling, & Blue, 2013). We understand the TACT trial as field of social practice (Pool & Geissler, 2005) in which assemblages of technology, health workers and patients, policy prescription and training practices, and medication come together with social relationships and expectations of care (Cohn, Clinch, Bunn, & Stronge, 2013; Collier & Ong, 2005).

Our main argument revolves around our interpretation of the TACT trial as a project that intertwined political, social and biological processes as it sought to intervene in the socio-material relations of health workers, patients and medicines (Prince, 2012; Rose, 2007). It relates therefore to the literature on bio-politics, and to the concerns with the ways in which biological knowledge and definitions have become central to our conception of self but (more importantly here) also become the sites upon which governance, negotiation and debate take place (Prince, 2012; Rose, 2007). Through our analysis, the TACT trial emerges as site of governmentality. We show how it sought to install a particular regime of practice (Dean, 1999 (2010)) through attempts to craft new subjectivities among health workers (through learning and practicing technologies of the self); by putting in place mechanisms of surveillance and establishing the role of the mRDT as a technical mode of truth telling (Foucault, 2011).

Like others drawing on Foucault and bio-political frames to analyse health in East Africa, however, we do not see this intervention as a perfectly implemented, ‘seamless bio-political disciplining project’ (Geissler, Rottenburg, & Zenker, 2012). Rather we show how the trial's ability to govern medicine and extend power into the facility was limited, and we explore how the TACT trial, as a form of governmentality, played out in spaces in which the relationships between health workers, patients and medicines were also shaped by other forms of care and moralities (Marsland & Prince, 2012). Our work, therefore, speaks to the literature on the locally embedded nature of decision-making around treatment that takes into account the results of technical investigations, but is also contingent upon knowledge about a patient's economic and social situation; relationships between clinicians and patients; and local possibilities for treatment (Armstrong & Hilton, 2014; Mol, 2003; Mol & Law, 1994; Street, 2011). We show that while the trial envisaged the mRDT as a tool to be adhered to, local knowledge often challenged its significance in the clinical encounter.

## The TACT trial

**Figure 1.**
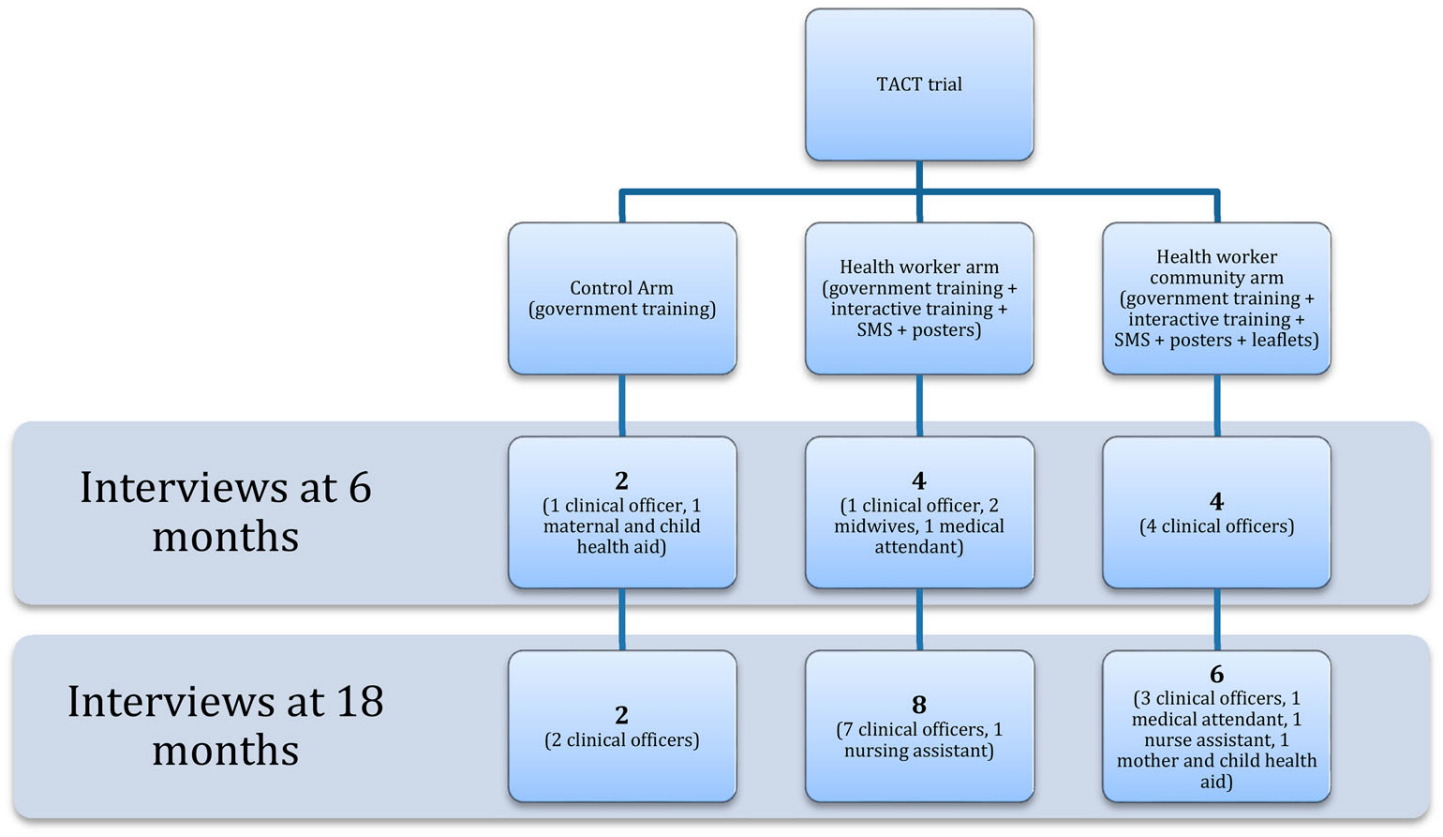
TACT trial – interviews.

The TACT trial was constructed to evaluate the effect of small group, participatory training and the use of communication tools on the uptake of mRDTs and on the prescription of ACTs in low-level public sector facilities in Tanzania (Chandler et al., 2014). It comprised three arms: the control arm, the health worker arm (hereafter HW arm) and the health worker patient arm (hereafter HWP arm). The control arm was based entirely on the Tanzanian Ministry of Health's two-day mRDT didactic, classroom-based training programme that was being implemented at the time. It relied upon a learner manual and practical guide (a job aid) with a short facility-based practical session. In the HW arm and HWP, health workers also received this government training. In addition, both groups were provided with three extra sessions that were described as teaching participants how to adapt to using mRDTs, how to use an mRDT with confidence and how to sustain these changes in practice. It provided copies of research papers about mRDTs; encouraged health workers to experiment with mRDTs by observing the impact of restricting prescriptions of ACTs to those with mRDT-positive results and underscored the use of mRDTs as part of the practices of a modern health worker. The training sought to develop communities of practice (Wenger, 1998) and emphasised shared learning. It also developed health worker skills of managing patients in the process of instituting this change (Figure 1).

Following the training in the HW and the HWP arms of the trial, a series of SMS texts were sent to reinforce the messages provided in the training with the aim of motivating health workers by providing feedback on their performance, and messages that encouraged the use of mRDTs. In addition, both HW and HWP arms received a poster setting out that fevers encompass more than malaria and that they should trust the test. In the HWP arm of the trial a pictorial, story-based patient leaflet was provided, setting out that mRDTs are available; why mRDTs are trustworthy; encouraging patients to experiment in not taking anti-malarials after negative test results; and that health workers should give information to patients. This was expected to provide a model for good practice, and give support to health workers in communicating results and treatment decisions to patients who suspected they had malaria.

## Research methods

Embedded within the TACT trial was a qualitative study to explore the health workers’ response to the training and the intervention as a part of the overall evaluation of the trial. The research comprised 26 face-to-face, in-depth interviews with health workers at six months (*n *= 10) and at 18 months after the trial began (*n *= 16). The majority of the interviews were conducted six months after the trial had closed, allowing space for critical reflection of the trial and enabling interviews to be undertaken when the trial no longer had any formal power over the participants. At both time points, the majority of interviews were undertaken in the intervention arms of the trial (HW and HWP), reflecting the researchers’ interest in providing an in-depth analysis of the relationships between the different elements of the trial and the shaping of practice.

The research participants were identified through the TACT trial records. The inclusion criteria were health workers who regularly prescribed anti-malarial treatment at the health facility and who had attended training on mRDTs in 2011. Participants were selected purposively to incorporate different cadres and include good and poor performers in terms of uptake and adherence to mRDTs. Neither interviewer nor interpreter was aware of who were better or worse performers during the interview and none of the participants were interviewed at both 6 and 18 months. Participants were invited to participate by the TACT trial field research team, and asked for written consent before the day of the interview and verbal consent and agreement to tape recording on the day.

The interviews were mostly conducted in English or in a mix of English and Swahili (with the exception of one in the health worker arm at six months, conducted entirely in Swahili) by interviewers who were not members of the TACT trial team. This was a deliberate choice on the part of the interviewers to enable the interviewees to express themselves as effectively and fluidly as possibly. A bilingual social scientist (a member of the TACT study team) was present at each of the interviews to provide a meaning-based translation when the need arose. The translator was given the space to check her translation with the informant whenever she was unsure of the meaning of their conversation.

The interviews began with introductions that underscored the importance of speaking freely about the test and the trial, and that the interviews were not a form of assessment. The first set of questions revolved around the most significant change that the health workers had experienced in the dispensary in the recent past, a method developed to generate detailed qualitative descriptions of interventions in low-income settings (Dart & Davies, 2003). The interviewer had a list of additional prompting questions about what the changes were, how they were made and why they were important to the participant (Dart & Davies, 2003). This was supplemented with further questions about the health workers’ experiences of and practices around the different elements of the trial (the test, the training, the SMS messages, the poster and the leaflet). It ended with a question about whether or not they trusted the mRDT and a series of questions about specific occasions when they had thought that the test result was wrong and how the situation had been managed.

At six months, only the English language translations were transcribed, a considerable limitation of the study. At 18 months the interviews were transcribed and translated in full. The initial analysis of the data was undertaken by the interviewers, under the supervision of an anthropologist to provide a descriptive account of the impact of the different elements of the trial. A second round of analysis was then performed, relying on both theory-driven and inductive analytic strategies that form the basis of this paper.

### Ethical clearance

The study was approved by the Ethical Review Boards of the National Institute for Medical Research in Tanzania and the London School of Hygiene and Tropical Medicine (LSHTM) (NIMRlHQ/R.8cNol. 11/24 and #5877, respectively). The trial was registered with clinicaltrials.gov (Identifier # NCT01292707) and was subject to external monitoring from LSHTM to ensure adherence with the protocol.

## Results and discussion

### Narratives of change: aligning with evidence-based medicine

Most health workers interviewed at both 6 and 18 months described a process of care-giving in their clinics as one that was expected to end in the prescription of medicines or on occasion the referral of patients to another clinic or hospital. Explaining how mRDTs impact on these practices, health workers pointed to shifts in prescribing patterns, switching treatments, and referring patients for further tests. Few described the mRDT as enabling them to make better diagnostic decisions (often described of as thinking of further problems). Of those that did, one described the introduction of mRDTs as having put pay to a trend in which patients failing to recover from their illness would return multiple times to a clinic to be treated each time with malaria medication.

Health workers across all the arms of the trial were well-versed in the political economy of the mRDT, and the benefits that they had for government expenditure. During the interviews, health workers often aligned themselves with these aims of the test's introduction as it was set out in the training, in some cases perhaps perceiving that this was what was hoped for by the interviewers. Past practices around the provision of care and medication for patients cast as both random and wasteful and a threat to the efficacy of the drugs. Some health workers developed the narrative of the training, stating that previous practices were dangerous and had ‘destroyed’ patients’ lives. Across all arms of the trial at both 6 and 18 months, the description of the use of the new diagnostic tool was, in contrast, presented in language that foregrounded global and national policy concerns and discourse: that they are economic, (disease) specific and proper, with the use of the mRDT emerging as a moral issue.

R: Now we treat patients thoroughly. We don't prescribe using clinical signs and symptoms as we were previously.

I: And you are happy with that?

R: Yes, because we were using a lot of medicine but now we just use a small amount of Alu [ACT] and this is an economic benefit for our country for all sections all health facilities using that procedure. We were using a lot of drugs unnecessarily.

Clinical Officer, Control arm at 18 months

Yet, the narrative in the training did not simply work as a new morality in the practice of prescribing medicines. Health workers were able to map their role in the governance of medicines. The narrative below, for example, makes connections between their clinical history-taking, using an mRDT, making decisions around medicines and the activities of the government. It also served to make practices in the clinic an issue of saving and redistributing the government health budget.

Previously it was, when a patient came to the facility, if they had a fever, we would prescribe an antimalarial. We would not discuss their symptoms with them or ask any questions. But now we are not using them so much. If you go back and look at our ordering system we were using the anti-malarials and then we were ordering a lot of drugs. When they were finished and we ordered more and then they were finished. But now, we have discovered that we order very few antimalarial drugs. And this will help in the government budget so that the budget that was used for these drugs can go somewhere else.

Clinical Officer, HWP arm at six months

### Practicing change: changing the (professional) self

Fashioning previous practices around the diagnosis of malaria in the clinics as random, wasteful and dangerous and the new way of diagnosing malaria as scientific and modern was part of a process in which the TACT trial sought to fix the interpretation of the relevance and position of the mRDT in the everyday practice at the health facilities. In so doing, the training presented the mRDT as a representation of modern biomedicine and those who used it as modern clinicians in opposition to traditional doctors who continued to use signs and symptoms. The TACT training supplemented this with a number of journal articles that set the mRDT in its intellectual, scientific context. Yet, health workers in the intervention arms of the trial were not expected to simply learn and read about biomedical knowledge. The intervention's conceptualisation rested on the notion that early adopters of mRDTs were able to *experiment* with restricting access to malaria medication on receipt of a negative mRDT result (Chandler et al., 2014). Under the trial, health workers were encouraged to reproduce the new knowledge that they had learned in the training within their health facilities: new rules were expected to be internalised through new regimes of observational practices when a particular medical gaze (and economic framework) was turned on both themselves and on their patients (Foucault, 1994). Health workers reported that experimenting with the test by restricting their own access to malaria medication and observing whether they would recover without taking an ACT had a powerful effect on their behaviour. For example,

I was feeling very feverish and then I decided maybe to teach myself. I asked the other health worker to test me with the RDT so that I could see how it reacted. So, I was tested and it was negative and I said, ‘I have to follow what the training taught so that I can see if it works on me or not.’ So, I said, ‘so long as it is negative then I will just use the amoxicillin and pain killers,’ something like that. So, I rested for five days and the fever went and from there I said, ‘No, I should not be taking the antimalarial,’ and I have not taken an antimalarial since then.

Clinical officer, HWC arm at six months

Although health workers did not always define this as a successful process, these changes in and observations of their own practices were mostly articulated as positive experiences that powerfully reaffirmed the lessons learned in the training, changing the ways in which they understood their own sickness and use of malaria medication.

### Incorporating the test into practice: the care value of drugs

While the TACT trial (following government policy) made demands that the mRDT become the sole tool upon which a decision about treatment for malaria would be made, the test results were often incorporated into a broader decision-making process which also relied on other sources of knowledge. Health workers, while keen to use the test, also incorporated concerns about patients who failed to recover after several visits to the health facility, patient's interpretations of their illness, clinical signs and symptoms of malaria and a history of patient's movement (into an area with high rates of malaria) in the diagnostic processes. They also described taking into account patients’ socio-economic status

The patient did not want to be referred because of her responsibilities at home. I gave her an ACT, an anti-vomiting drug and oral rehydration salts to help her as she told me that husband is living far away, she has a small child and no money. (Clinical officer, Control arm at six months)

These considerations had the potential to overwhelm the position of the test in the clinic as the tool upon which a decision about malaria treatment would be made and patients with a negative test result would be treated with an ACT.

Health workers who continued to rely upon practical knowledge in order to make their diagnosis also described times when they had been concerned that the test had failed due to human error. In these cases, issues around who had conducted the test, whether there was enough buffer, whether patients had taken other medicine that might affect results, whether there might have been too much blood in the test, or if the test read ‘neutral’ shaped the role of the test in the decision-making about treatment, with some patients with a negative test result being treated with malaria medication. Importantly, this was not considered by one health worker as an example of not trusting the test per se, but rather as reflecting the uncertainties of the practices around the test in the clinic:

CO: Personally, I have used an mRDT to test for malaria. It was negative and so I went to have a test using microscopy. They told me that I had malaria with a 3+ parasite load but I didn't take any antimalarial so I think it is accurate.

I: Have you ever thought that may be the test is incorrect?

CO: Yes, it happened when a boy of two years came in with vomiting and fever. I was not the person who conducted the test (my colleague did it) and I looked at the test I couldn't agree with the results.

I: What did you do?

CO: I just gave him an anti-malarial.

I: Why do you think it was negative if you thought he has malaria?

CO: I thought that maybe there had not been enough buffer. I say maybe because I was not there when he conducted the test.

I: Can you think of the reasons why the test might be incorrect sometimes?

CO: May be someone can put few drops of buffer, I think that is the most (common) thing I can think of.

Clinical Officer, HW arm at 18 months

In some cases, the test emerged as being of most value at the beginning of a consultation, enabling the health worker to rule in or out malaria at this point. Patients would then be assessed for other causes of illness and, if none could be found, then the health worker could return to a diagnosis of malaria. While the efforts to seek an alternative diagnosis and treatment accords well with a normative model of biomedical care, descriptions of providing patients with medication demonstrated that it did not simply work as a means of providing a treatment for an identifiable disease. Health workers described a process of replacing malaria medicine with other drugs with no emphasis laid on making a diagnosis (‘Whereas previously I could give anti-malarial to every patient with fever, cough, or body pain, now if the mRDT is negative I will go for an antibiotic.’ Clinical Officer, HW arm at 18 months). Others used less powerful medication, describing how medicines had to be provided in order for care to be experienced.

When the patient comes even if it's negative, it has to be dealt with carefully. I give placebo, I can just prescribe paracetamol and B-complex or magnesium so that she or he feels she or he has come from hospital. For example, if we'll do as we were taught in the seminar it has to be – if it is negative no need to prescribe anything – that will really bring conflict especially to us in rural areas. That is that's why we are trying to prescribe placebo like B-complex or paracetamol because such a person is negative and there is no need to give any other drug – unless otherwise she or he is presenting with other symptoms you can either consider piriton and tell him/her how to take it.

Clinical officer, HW arm at 18 months

In the face of their new restrictions around malaria medicines, health workers also became creative, using innovative strategies to enable patients to be satisfied with their care while they also adhered to the policy of the intervention. One clinical officer, interviewed at 18 months, refused to replace ACTs with other medicines, repeating and extending concerns raised by other health workers that using medicines unnecessarily can ‘destroy’ a patient's health. He described how he combined the test, health education and the gift of an empty pill container to foster satisfaction and contentment in his patients who tested negative for malaria, something he considered a critical element of care-giving. Yet, it was not simply the changes learned in the training that shaped these new care-giving practices; the receipt of an SMS could work powerfully to mediate relationships between health workers, patients and (malaria) medicines.

### Adhering to the test: SMS as a form of surveillance

The SMS technology used by the trial was designed to impact on provider practice from a distance (cf. Rose, 2007) and extend the narratives of the training to underpin new practices associated with it in the clinic. Health workers reported that these texts worked well by framing the mRDT in the knowledge that they gained in the training and reconnecting them with the facts that they were presented with – that mRDTs were trustworthy and that they should use them on all patients with fever and to direct their decision-making about when to prescribe an ACT. Many health workers described the SMS as having an immediate effect on their practices within the clinic. This was often not simply a process of remembering lessons learned but one in which health workers felt that the SMS provided them with power enabling them, for example, to stand firm in the face of patient demands for medication.

Ok, the test said negative, but the patient was complaining a lot. Then I tried to counsel him but he said, ‘Usually when feel like this I take malaria medication and I get better but without the drugs I am not getting better.’ He did not want to leave. He just sat there insisting I should write a prescription for a malaria medication. Then, the message came through my phone and I read it. Then I kept insisting to the patient that I wouldn't give him the medicine.

Clinical Officer, HW arm at 18 months

While the SMS could be interpreted as conferring power onto the health worker, when health workers wished to act against the results of the test, its significance in the clinic could shift. The combination of an SMS and test result could become a powerful challenge to the health worker's decision-making about the best course of action for the patient, with the power resting with the technology and with those who were providing the tests and the messages rather than with the health worker. At these moments, the text messages emerged less as a helpful reminder or device to empower health workers as they managed patients’ hopes around medicines and more like an unwanted form of surveillance, that made health workers consciousness of the fact that their interaction with mRDTs and malaria medication were under scrutiny by those beyond the clinic. This reconfigured the mRDT as a tool that made significant demands, creating conflict between older moralities of providing care and newer ones of adhering to results.

CO: Sometimes the SMSs really surprised me. I have treated a patient who tested negative with an antimalarial but when I receive a text message that saying that I should treat the patient according to the test result and not according to my feeling, then I feel that I have done something wrong. Sometimes the patient comes and you test and if it is negative you give antibiotic for five days. But, after five days the patient may come back and say they are still not feeling well so at that point … . you know the patient is a human. Then the patient may come like three or more times again in the same situation and you test and it is negative and so you don't give an antimalarial. Then, the time that you do give an antimalarial is the one when you receive an SMS saying ‘treat according to the test result’ and then you feel like these people are watching me. I remember an example of a woman who was sick. She had a headache and a fever but the test showed no malaria and I gave her antibiotic for the cough and paracetamol for her fever. After a week, she came back again and I tested her and she had no malaria. I told her that she should use painkillers and come back after two days. Again, she came back with a fever and headache and she asked me to give Alu because she is not recovering.

I: When you received the message to treat according to the test results did that make you change what you were doing?

CO: I agree that I have changed but sometimes it is very difficult. It takes me a long time to give a patient [with a negative test result] an antimalarial.

Clinical Officer, HW arm at 18 months

Of significance in this discussion is the way in which the clinical officer understands the patient's desire for medicine as part of their humanity, something that always adhering to the test result has the potential to undermine.

### Adhering to the test: posters, leaflets as the justification of decision-making

In the HW and HWP arm of the trial, the health workers described the use of the poster (HW and HWP arm) and the leaflet (HWP arm only) as echoing the work of the SMS to situate the test in the policy framework, bringing facts about the accuracy of mRDTs, declining rates of malaria in Tanzania and the correct way to treat patients (through alternative medication and referral) directly into the clinic. For many health workers, the posters and the leaflets were interpreted as effectively providing information about the change of practice in a clinic, and introducing patients to new forms of treatment. Some reported that they also relied on them to refresh their own memories of the correct way to conduct the test. For other health workers, however, concerns with patients’ negative response to or suspicion of the introduction of the mRDT, the leaflets and posters were also being used to provide power to the test results and the health worker by locating the relationship between the test, the health worker and the patient within government policy (‘At the beginning the patients thought the tests are ours so we were telling them to read the poster, that the tests were brought by the government.’ Clinical Officer, HW arm at 18 months), effectively demonstrating that there was a powerful external force shaping practice around diagnosis and treatment for malaria within the clinic. Yet, the leaflets and the poster did not always work in convincing patients about the need to use the tests and health workers developed other techniques in the clinic as they managed the implications of the test results.

So then I had to use [a] kind of trick that this is the law that is being enforced, that when this device is positive I have to record that it is positive and then I have to give the antimalarials. But for now this one doesn't say that it is positive, then [if] I have to record that I have given you antimalarial so it's kind [of], I'm going to be sued. So, I just try to make the patient understand. So I even told the person, those who are bringing the test are the ones giving the drugs, so they will question me and I won't have the way to explain to them why this happens this way.

Clinical Officer, HWP arm at six months

The clinical officer above had access to a number of materials that could have helped him to describe the benefits of parasitological testing to the patient. Yet, his description of the encounter in the clinic shows how he was left unable to find the words to convince the patient that it was logical that he was relying on the test to make a decision about treatment. Instead, he presented the impossibility of acting against the test to the patient, resituating its results as a ‘matter of law’ and the role of the health worker as one of acting on government policy, as an agent of the government rather than a health worker with an obligation to provide care for the patient.

## Conclusions

In this paper, we have analysed the TACT trial as a project that sought to intertwine social processes (the creation of new identities and relationships between health workers and patients); political issues (about the relationship between health workers and the government) and biological processes (of the treatment and management of diseases) as it sought to set the role mRDT in public sector clinics. In our analysis, the TACT trial's processes of self-observation and experimentation with mRDTs have been presented as a technology of the self, regulating the use of ACTs as health workers created knowledge about their own bodies and those of their patients (Foucault, 1990 [1985]). We have shown how the trial seems to have been most powerful as a form of governance (as described by Foucault in his later work) as it bundled together these techniques of the self with technologies of surveillance (SMSs, posters and leaflets) to underscore the role of the test as a mode of technical truth telling (Foucault, 2011).

The TACT trial did not always create a substantial shift in these socio-material relations in the clinics. The test could be incorporated it into practice as part of a decision-making process that also drew on other, locally produced knowledge and the mRDT's role in the clinical encounter could be undermined. It seems likely that these processes are also underway in other clinics in Tanzania, with results from routine clinical practice in Tanzania showing that mRDTs do not dominate as a means of decision-making around malaria (Bruxvoort et al., 2013). At certain moments, however, processes of self-regulation and surveillance appeared to be very powerful in the clinic, challenging the social and moral order primarily around the hopes and sometimes demands for care that is encapsulated in the provision of medicine. Health workers who felt unable to prescribe ACTs in the ways that they wanted described being left unable to respond to the humanity of patients or having to trick them into accepting new practices. This echoes other work on self-regulation in other institutional settings in Africa (notably in schools and among humans rights activists) in which attempts to create new forms of subjectivity in some social actors are entangled with practices of objectification in others (Englund, 2006).

In this way, the mRDT could appear less a tool that could support a diagnostic process, and more a tool enabling an economic intervention to save precious medical supplies, enabling governments to exert considerable control (from a distance) in places that have traditionally been hard to reach. In addition to their ability to reduce costs associated with treatment in areas of low prevalence of malaria, it could be that it is this (potential) role of the mRDT as part of a package of measures to render peripheral spaces governable that is part of its continued attraction to these tests despite the fact that their impact on health outcomes has yet to be demonstrated (Beisel, 2014; Odaga et al., 2014).

Overall, the experience of introducing mRDTs in the different arms of the TACT trial revealed the contingent nature of these tests as a medical tool; that rather than being an autonomous force (as is often suggested in policy discourse) other objects, processes and relationships shape their practical significance in the health facilities. Over the next decade, a number of other rapid diagnostic tests are likely to be introduced in low-income settings and it seems likely that this will be similarly shaped by the contexts in which they are placed. While the use of other diagnostic tests and processes may not challenge obligations between health workers and patients in the same way as an mRDT (as their role may not primarily be about reducing the use of a particular medicine), their introduction may well shift processes of care-giving, enabling different configurations of social and moral order to come into being (Schubert, 2011). If mRDTs and other tests can be incorporated into practice in a way in which the judicious use and interpretation of test result could complement clinical decision-making, it may increase the efficacy of treatment without undermining current care practices. The resolution between the more flexible and more dogmatic interpretations of the mRDT that we have shown here may come with improved training of health workers, and through better resourcing of health facilities, enabling well-trained staff in well-resourced clinics to use test results critically and in a way that enhances their duty of care.

## Strengths and limitations

This qualitative study took place alongside a cluster controlled trial to provide an understanding of the broader impacts of introducing rapid diagnostic tests on care-giving processes. This adds to the existing literature describing the immediate challenges and opportunities presented by mRDTs to health workers in Tanzania and elsewhere (Chandler et al., 2008, 2012). Our sampling strategy ensured we included a range of participants in terms of their use and uptake of the tests under the TACT trial at two different time frames. Our approach however, is limited in its use of a translator and interviews that rely on the participants’ own accounts of their practices, as it is possible that their presentation of their use of the test was framed in a particular way during the interviews. Moreover, these interviews could not take into account patient perspectives on the introduction of the test and patient interpretations of the change in practice around malaria. A different approach that relied upon participant observation, for example, would have permitted an analysis of the role of mRDTs in relation to a broader range of practices in the dispensaries, and how negotiations between health workers patients are managed over the uptake and adherence to test results.
